# Prescribing cascades in community-dwelling adults: protocol for a systematic review

**DOI:** 10.12688/hrbopenres.13345.1

**Published:** 2021-07-13

**Authors:** Ann Doherty, Frank Moriarty, Fiona Boland, Barbara Clyne, Tom Fahey, Seán Kennelly, Emma Wallace

**Affiliations:** 1 Department of General Practice, RCSI University of Medicine and Health Sciences, Dublin 2, Ireland; 2 School of Pharmacy and Biomolecular Sciences, RCSI University of Medicine and Health Sciences, Dublin 2, Ireland; 3 Data Science Centre, RCSI University of Medicine and Health Sciences, Dublin 2, Ireland; 4 Department of Medical Gerontology, Trinity College Dublin, Dublin 2, Ireland; 5 Department of Age-related Healthcare, Tallaght University Hospital, Dublin 24, Ireland

**Keywords:** systematic review, protocol, prescribing cascades, adverse drug reactions, polypharmacy

## Abstract

**Introduction:** Internationally, health systems face the challenge of managing a growing ageing population living with multimorbidity and polypharmacy. Potentially inappropriate prescribing is common among patients with polypharmacy, increasing the risk for adverse drug reactions (ADRs). Several prescribing indicator sets exist to improve prescribing and reduce potentially inappropriate prescribing, but do not address prescribing cascades. Prescribing cascades occur when a medication is prescribed to treat an ADR to another prescribed medication, whether intentionally or unintentionally, and constitute an important area to consider when characterising problematic polypharmacy. This is a protocol for a systematic review examining prescribing cascades in community-dwelling adults.

**Methods:** The review will be reported adhering to the Preferred Reporting Items for Systematic Reviews and Meta-analyses (PRISMA) guidelines. A systematic search of Medline (Ovid), EMBASE, PsycINFO, CINAHL and the Cochrane Library will be conducted from inception to March 2021, using a predetermined strategy. Grey literature will be searched using Open Grey, MedNar, Dart Europe, and the Turning Research Into Practice (TRIP) databases. No restrictions will be placed on language or publication year. Inclusion criteria are: population - community-dwelling adults (≥18 years), including those in residential or nursing homes; risk - prescription medication with the potential to cause side effects; outcomes - initiation of a new medicine to ‘treat’ or reduce the risk of experiencing an ADR. Prospective and retrospective cohort studies, case control and case series studies will be included. Two reviewers will independently screen titles and abstracts; studies meeting inclusion criteria will undergo independent full-text screening by two reviewers.  A narrative synthesis will be conducted. Study quality will be independently assessed using the relevant Joanna Briggs Institute Critical Appraisal Checklist.

**Discussion:** This systematic review will identify examples of prescribing cascades for community-dwelling adults and contribute to developing an evidence base regarding such cascades.

**Registration: **PROSPERO [ CRD42021243163, 31/03/2021].

## Abbreviations

ADR: adverse drug reaction; ATC: Anatomical Therapeutic Chemical; MeSH: Medical Subject Headings; NSAID: non-steroidal anti-inflammatory drug; PRISMA: Preferred Reporting Items for Systematic Reviews and Meta-analyses; PRISMA-P: Preferred Reporting Items for Systematic Reviews and Meta-analysis Protocols; STOPP: Screening Tool for Potentially Inappropriate Prescriptions; TRIP: Turning Research Into Practice; WHO: World Health Organisation.

## Introduction

Caring for older people with multiple chronic medical conditions, known as multimorbidity, is now the greatest challenge faced by health systems internationally. However, to date the vast majority of research and clinical guidelines have focused on single diseases, whereas in reality most patients have multimorbidity, necessitating multiple treatments ^1^. Treatment burden for older people has increased substantially. In Ireland, over 60% of those aged ≥65 years are taking five or more prescribed medications (known as polypharmacy) and 20% are taking 10 or more ^2^. Medications provide many therapeutic benefits but these must be balanced against the potential for patient harm. Potentially inappropriate prescribing is common among older adults with polypharmacy and increases the risk for adverse drug reactions (ADRs) ^3^.

However, the challenges posed by multimorbidity, polypharmacy and potentially inappropriate prescribing are not restricted to older adults alone and can affect people of any age ^1^. In absolute terms multimorbidity is more prevalent in those aged 65 years or younger and has been shown to occur some 10–15 years earlier for those people living in socioeconomic deprivation than for those living in more affluent areas ^4^. A higher prevalence of multimorbidity among younger adults is likely to increase the risk for inappropriate prescribing and ADRs among those under 65 years of age also.

While medication counts are the greatest predictor of medication-related harm, simple counts of medicines cannot account for clinical appropriateness ^5– 8^. Several prescribing indicators sets have been developed to characterise overall prescribing quality, including explicit prescribing indicator sets such as the Screening Tool for Potentially Inappropriate Prescriptions (STOPP) and Beers criteria, as well as implicit measures such as the Medication Appropriateness Index ^9– 11^.

However, much less is known about other causes of problematic polypharmacy such as prescribing cascades, which are not captured in existing explicit or implicit prescribing indicators. A prescribing cascade can occur when a prescribed medication causes an ADR ^12– 14^. If the ADR is misinterpreted as a new medical condition and results in the subsequent prescription of another medication, an unintentional prescribing cascade occurs ^15^. An example of an unintentional prescribing cascade is the prescribing of a loop diuretic to treat lower extremity oedema caused by calcium channel blockers ^16, 17^. Intentional prescribing cascades occur when the ADR is recognised and attributed to the first medication and a subsequent medication is intentionally prescribed to combat this ADR or is prescribed at the same time as the first medication in order to prevent it ^15^. An example of an intentional prescribing cascade is the prescribing of a proton pump inhibitor (PPI) to minimise the gastrointestinal effects of non-steroidal anti-inflammatory drugs (NSAIDs).

Prescribing cascades may occur at any age but may be more prevalent among older adults. ADRs can be difficult to recognize in older people as they often present with nonspecific symptoms, such as falls, fatigue or constipation, all of which have several potential causes ^18^. Therefore, it can be difficult to recognize whether a new symptom is due to an ADR in an older person with multimorbidity or because of other underlying medical conditions. Failure to recognise an ADR may then result in a prescribing cascade, thus inadvertently continuing the patient’s exposure to the culprit medication causing them harm, and additional potential risk from the newly prescribed medication ^12^.

Prescribing cascades may also be further dichotomised as either appropriate (benefits outweigh risks) or problematic (risks outweigh benefits) ^15^. Central to any assessment of the appropriateness of the cascade is the inclusion of the patient within the assessment, with particular consideration given to whether the initiation of the cascade aligns with the patient’s goals and their awareness of the potential long-term risks of the cascade ^15^.

Nevertheless, prescribing cascades are under-researched as highlighted by a previous scoping review where only 10 original investigations and seven case reports of prescribing cascades were identified ^19^. This scoping review adopted a broad perspective and sought to systematically describe the resources available to prevent, detect and reverse prescribing cascades. However, studies that did not report a strategy to prevent, identify or reverse a prescribing cascade were excluded from the review. In their review, Brath and colleagues argue that it is likely that some clinically relevant prescribing cascades have yet to be identified or characterised ^19^. The review authors found that the majority of included studies were published within the last two years of their search period (2015–2017), in spite of the phenomenon first being described more than 20 years ago. This current systematic review will build upon the work of this earlier scoping review and aims to identify and collate an exhaustive list of published prescribing cascades specifically in community-dwelling adults. Specifically, this study seeks to answer the following question: Which medications result in prescribing cascades experienced by community-dwelling adults?

## Methods

The systematic review protocol has been prepared in line with Preferred Reporting Items for Systematic Reviews and Meta-analysis Protocols (PRISMA-P) guidance ^20^ and has been registered in PROSPERO [ CRD42021243163]. In the event that protocol amendments are necessitated, a description of the change required and the rationale for change will be provided in conjunction with an amendment date. The PRISMA-P checklist is available as extended data ^21^. The systematic review will be reported as per the Preferred Reporting Items for Systematic Reviews and Meta-analyses (PRISMA) guidelines ^22^.

### Eligibility criteria

***Participants/population.*** Inclusion criteria: Community-dwelling adults (≥18 years), including those living in nursing homes and residential care in the community.

Exclusion criteria: Those under 18 years, hospital inpatients, and those attending hospital Emergency Departments (EDs).

***Risk.*** The risk of interest will be the prescription of any medication which has the potential to cause a side effect that results in the prescription of further medication. Details of the initial medication prescribed, including the therapeutic indication and, where available, the side effect resulting from the initial medication, will be recorded. Medications will be categorised according to the World Health Organisation (WHO) Anatomical Therapeutic Chemical (ATC) classification system. In cases where ATC codes are not reported within the study text, ATC codes will be assigned at the appropriate level e.g. 5 ^th^ level where the chemical substance name is reported or 4 ^th^ level where the chemical subgroup is reported etc.

***Outcome.*** Prescribing cascade defined as the initiation of a new medicine to ‘treat’ an adverse reaction to another medication (unintentional cascade) or to reduce the risk of experiencing an adverse reaction to a medication (intentional cascade).

***Types of studies.*** Prospective and retrospective studies, case control and case series studies will be included. Case reports will be excluded. 

Studies identified during full text screening which report on a prescribing cascade will be included irrespective of whether the primary aim of the study was to identify or evaluate a prescribing cascade or not.

***Setting.*** Primary care and community settings including ambulatory care settings.

### Search strategy

The following databases will be searched: Medline (Ovid), EMBASE, PsycINFO, CINAHL and the Cochrane Library from inception to March 2021. There is no medical subject heading (MeSH) for prescribing cascades. Databases will be searched using combinations of keywords to capture concepts related to incremental, sequential or cascading prescribing. MeSH terms that relate to ADRs will also be included within the search strategy to capture potential prescribing cascades that have yet to be identified and characterised.

Grey literature will be searched using Open Grey, MedNar, Dart Europe, and the Turning Research Into Practice (TRIP) databases. The search will be supplemented by forward and backward citation searching of retrieved articles. No restrictions will be placed on language or year of publication. The search strategy for MEDLINE (Ovid) is available as extended data and will be adapted for the different databases ^21^. The search strategies will be developed in consultation with a librarian experienced in systematic review searching.

### Data management

Search results will be exported to Endnote X9 reference management system. Following this, Covidence will be used to screen abstracts according to inclusion and exclusion criteria and manage selected articles. Data extraction will be conducted in Microsoft Excel using a standardised proforma (available as extended data) ^21^.

### Study selection

Titles and abstracts will be independently screened by two reviewers (AD and EW) to identify studies that potentially meet inclusion criteria and to remove ineligible and duplicate titles. Studies that do not meet inclusion criteria will be excluded. Where it is unclear whether a study meets the inclusion criteria it will be selected for full text review. Disagreements will be managed by consensus or via a third reviewer where necessary. Additional data will be sought from authors where necessary. A PRISMA flow diagram will be used to indicate the flow of information through the different phases of the systematic review.

### Data extraction

Data will be extracted by two independent reviewers (AD and EW) using a standardised data proforma ^21^ on;

•   Author and year

•   Study setting

•   Study population

•   Type of study

•   Outcome (prescribing cascade)

◦ How outcome was measured e.g. patient self-report, routine data etc.
◦ Details of the initial medication(s) prescribed (medication class or individual medication including ATC code) and how recorded (e.g. dispensed medication, prescribed medication etc.)
◦ Details of the medical condition(s) for which the initial medication(s) was prescribed
◦ Type of adverse reaction(s), where reported (e.g. symptoms or diagnoses resulting in prescription of subsequent medication)
◦ Details of new medication(s) prescribed (medication class or individual medication including ATC code)
◦ Where relevant, frequencies/percentages of participants prescribed new medications

•   Contextual and systems-based factors which may influence prescribing (where available) e.g. demographics, polypharmacy, inappropriate prescribing, recent hospitalisation etc.

•   Type of statistical analysis, where applicable (e.g. prescription sequence symmetry analysis, survival analysis etc.)

•   Confounders accounted for in the analysis (e.g. age, gender, deprivation, other medications, comorbidity etc.)

•   Quantitative measure of association between initial medication prescription and ADR occurrence and new medication prescription, where reported, such as the adjusted sequence ratio (ASR) for prescription sequence symmetry analysis

### Quality assessment

Studies that meet inclusion criteria will be included, irrespective of quality. Methodological quality assessment of included studies will be independently performed by two reviewers using the relevant Joanna Briggs Institute Critical Appraisal Checklist, dependent on study type ^23^.

### Strategy for data synthesis

We will narratively summarise the following for each prescribing cascade under the following headings;

•   Initial medication(s) prescribed to patient (medication class or individual medication including ATC code)

•   Subsequent adverse reaction(s) (symptom(s) or new diagnoses)

•   New medication(s) prescribed (medication class or individual medication including ATC code)

•   Study population demographics

•   Setting of care (e.g. residential vs non-residential)

•   Methodological approach to analysis (if appropriate)

•   Clinical importance of prescribing cascade (potential risk to patient)

•   Hypothesis generation data (case series)

### Study status

The search strategy for this study was developed in February 2021 with searches conducted in March 2021. Title and abstract screening commenced in April 2021, with full text screening expected to be completed by July 2021.

## Discussion

Prescribing cascades are a contributor to problematic polypharmacy but are not captured within the numerous prescribing indicator sets aimed at reducing the use of inappropriate medications. Known prescribing cascades include those resulting from commonly used medications such as antihypertensives, NSAIDs and cholinesterase inhibitors ^16, 17, 24, 25^. Calcium channel blockers, particularly dihydropyridine calcium channel blockers, have been shown to result in a prescribing cascade whereby the resultant lower extremity oedema is treated with loop diuretics ^16, 17^. A dry cough is a common side effect of ACE inhibitors and has been shown to result in the prescription of a cough suppressant and antibiotics ^24^. Cholinesterase inhibitors prescribed for older adults with dementia may precipitate urinary incontinence which may be interpreted by the clinician as part of the natural progression of dementia, resulting in the inappropriate prescribing of anticholinergic medications ^25^. In some instances, this prescribing cascade may be considered appropriate if the individual experiences a noticeable benefit in cognitive and functional status from the cholinesterase inhibitor ^15^. Prescribing cascades may also occur intentionally, for example the prescribing of a PPI to minimise the gastrointestinal effects of NSAIDs.

Multimorbidity and resultant polypharmacy increases the risk of experiencing medication-induced injury. Identifying medications that result in prescribing cascades will support clinicians to optimise their prescribing to benefit patient care. This systematic review will collate all available information pertaining to prescribing cascades that commonly occur in community dwelling adults and will thus contribute to developing an evidence base for this topic. In addition, an evaluation of the relative likelihood of various prescribing cascades, and their clinical importance, may help to prioritise cascades for attention or intervention. The identification of the ADRs most often implicated in prescribing cascades may guide prescribers to intervene to avert unintentional prescribing cascades in the future. Examining the study designs and analyses used to identify prescribing cascades may have implications for the design of future studies which seek to identify new prescribing cascades. It is intended that this systematic review will form part of a project which will provide GPs with a tool that they can use to support their prescribing decisions during multimorbidity consultations.

## Data availability

### Underlying data

No underlying data are associated with this article.

### Extended data

Open Science Framework: Prescribing cascades in community-dwelling people: protocol for a systematic review. Extended Data. https://doi.org/10.17605/OSF.IO/27SNR ^21^.

This project contains the following extended data:

- Data extraction proforma.xlsx (Excel proforma document with all headings under which study characteristics will be extracted)
- Medline (Ovid) Search Strategy.pdf (The combination of keywords and MeSH terms that will be used to search Medline and which will be adapted to other databases)

### Reporting guidelines

Open Science Framework: PRISMA-P checklist for “Prescribing cascades in community-dwelling people: protocol for a systematic review”. https://doi.org/10.17605/OSF.IO/27SNR ^21^.

Data are available under the terms of the Creative Commons Zero “No rights reserved” data waiver (CC0 1.0 Public Domain Dedication).
