# Supplementary material for: Prescribing cascades in community-dwelling adults: protocol for a systematic review
Source: HRB Open Res. 2021 Sep 21;4:72. Originally published 2021 Jul 13. [Version 2] doi: 10.12688/hrbopenres.13345.2 (PMC8529400; doi:10.12688/hrbopenres.13345.2)
Supplement: Peer review history

# Peer review: Reviewer response for version 2

**DOI:** 10.21956/hrbopenres.14614.r30342

**Published:** 2021-10-20

**Authors:** Nagham J. Ailabouni

**Affiliations:** 1 Clinical and Health Sciences, Quality Use of Medicines and Pharmacy Research Centre (QUMPRC), University of South Australia, UniSA, Adelaide, SA, Australia

Thank you for addressing my previous comments. I am satisfied with the changes the authors have made.

Is the study design appropriate for the research question?

Yes

Is the rationale for, and objectives of, the study clearly described?

Yes

Are sufficient details of the methods provided to allow replication by others?

Yes

Are the datasets clearly presented in a useable and accessible format?

Not applicable

Reviewer Expertise:

Geriatric pharmacy; deprescribing; multimorbidity

I confirm that I have read this submission and believe that I have an appropriate level of expertise to confirm that it is of an acceptable scientific standard.

# Peer review: Reviewer response for version 1

**DOI:** 10.21956/hrbopenres.14533.r29844

**Published:** 2021-08-17

**Authors:** Rachel D. Savage

**Affiliations:** 1 Women's College Research Institute, Women's College Hospital, Toronto, ON, Canada

This is a well-written systematic review protocol on a worthwhile topic – prescribing cascades. Results have clear implications (i.e., the potential to support deprescribing efforts) that can be used to reduce medication harms and improve patient safety.

**Abstract:**

1. In describing your population (“community-dwelling adults (≥18 years), including those in residential or nursing homes”), I was surprised that you included individuals residing in residential or nursing homes in your definition of community-dwelling. These populations are typically classified as distinct in scientific literature. Could authors please provide a justification for classifying as such?
2. If the volume of evidence supports it, stratifying results by setting (community vs. nursing home) would be helpful to guide preventive efforts given that contextual factors that contribute to prescribing cascades differ in these settings.

**Introduction:**

1. “In absolute terms multimorbidity is more prevalent in those aged 65 years or younger”. I agree with reviewer 1 that the statements about multimorbidity in <65 years are misleading given the use of the terms absolute and prevalent/prevalence. Do you mean to say that multimorbidity affects more younger adults because the population size is larger, is the prevalence of multimorbidity higher in younger adults, or both? The prevalence of multimorbidity increases with age; as a result, this statement is inconsistent with established evidence. 
2. In your example of an example of an unintentional prescribing cascade (i.e., CCB -> edema -> loop diuretic), I recommend also citing our study: Savage RD *et al.* Evaluation of a Common Prescribing Cascade of Calcium Channel Blockers and Diuretics in Older Adults With Hypertension. JAMA Intern Med. 2020 May 1;180(5):643-651 ^1^.

**Methods:**

1. Eligibility Criteria - What is the rationale for exclusion of those attending hospital Emergency Departments (EDs)? Adults using these services are often considered community-dwelling, and most prescribed medicines would be filled in community pharmacies. Also are EDs not considered ambulatory care settings (see Setting))? 
2. Risk – I agree that it would be useful to capture data on the therapeutic indication and the side effect; however, these details are often not available in health administrative data, and so would likely only be available in case studies. While therapeutic indication will not necessarily be available, studies using health administrative data may have selected certain patient cohorts (i.e., those with hypertension, etc) to study a particular cascade – this information would be relevant to capture. 
3. Data extraction – This is a comprehensive list but suggest adding – i) time to prescribing of drug B (i.e., time from initiation of drug A to initiation of drug B) – if recorded, this information is helpful to identify a relevant time window that providers can think about/be aware of when the side effect is likely to occur, and ii) variables considered to modify the association/effect, specifically sex and gender. This information again would be helpful in considering whether there are cascades which affect women vs. men, which can help inform targeted interventions.

**Discussion:**

1. I wonder about the inclusion of intentional prescribing cascades if the intent is to “support clinicians to optimize their prescribing to benefit patient care”? If cascades are started intentionally, clinicians have likely already carefully considered that benefits outweigh harms. If you wish to have an exhaustive list, I understand the desire to include both intentional and unintentional; however, I agree with reviewer 1 that stratifying results based on intent is helpful, since those that are unintentional are more amenable to deprescribing and improving patient safety.

Is the study design appropriate for the research question?

Yes

Is the rationale for, and objectives of, the study clearly described?

Yes

Are sufficient details of the methods provided to allow replication by others?

Yes

Are the datasets clearly presented in a useable and accessible format?

Not applicable

Reviewer Expertise:

Health services research in older adults, including studying prescribing cascades

I confirm that I have read this submission and believe that I have an appropriate level of expertise to confirm that it is of an acceptable scientific standard.

## References

[1] : Evaluation of a Common Prescribing Cascade of Calcium Channel Blockers and Diuretics in Older Adults With Hypertension. *JAMA Internal Medicine* .2020;180(5) : 10.1001/jamainternmed.2019.7087 10.1001/jamainternmed.2019.7087 PMC704280532091538

# Response

**Published:** 2021-09-07

**Authors:** Ann Doherty

**Affiliations:** Royal College of Surgeons in Ireland, Ireland

Dear Dr Savage,

 Thank you for reviewing our submission and for your thoughtful feedback. Below you will find our responses to the specific comments in turn. Text labelled 'Reviewer' are the comments of the reviewer. Text labelled 'Authors' are the responses of the authors.

**Reviewer 2: **In describing your population (“community-dwelling adults (≥18 years), including those in residential or nursing homes”), I was surprised that you included individuals residing in residential or nursing homes in your definition of community-dwelling. These populations are typically classified as distinct in scientific literature. Could authors please provide a justification for classifying as such?

**Authors**: We accept that those in residential or long-term care are typically classified as a distinct population within the literature. We have updated the inclusion and exclusion criteria of the manuscript to exclude those individuals residing in residential care or nursing homes.

**Reviewer 2**: If the volume of evidence supports it, stratifying results by setting (community vs. nursing home) would be helpful to guide preventive efforts given that contextual factors that contribute to prescribing cascades differ in these settings.

**Authors: **We agree with the reviewer that stratification of results by setting would be helpful. However, as we are now excluding individuals in residential care and nursing homes this will not be possible.

**Reviewer 2:** “In absolute terms multimorbidity is more prevalent in those aged 65 years or younger”. I agree with reviewer 1 that the statements about multimorbidity in <65 years are misleading given the use of the terms absolute and prevalent/prevalence. Do you mean to say that multimorbidity affects more younger adults because the population size is larger, is the prevalence of multimorbidity higher in younger adults, or both? The prevalence of multimorbidity increases with age; as a result, this statement is inconsistent with established evidence. 

**Authors: **We understand that our attempt to be concise has raised some queries regarding this section of the text. It was our intention to state that whilst multimorbidity is predominantly associated with older adults, there are often a large number of younger adults living with multimorbidity and who are thus consequently also at risk for polypharmacy and inappropriate prescribing. We have updated this section of text to state the predominant association between multimorbidity as outlined in the response to Reviewer 1 (Dr Ailabouni). 

**Reviewer 2:** In your example of an example of an unintentional prescribing cascade (i.e., CCB -> edema -> loop diuretic), I recommend also citing our study: Savage RD *et al.* Evaluation of a Common Prescribing Cascade of Calcium Channel Blockers and Diuretics in Older Adults With Hypertension. JAMA Intern Med. 2020 May 1;180(5):643-651 ^1^ .

**Authors: **We have updated the Introduction section to include the recommended citation, which appears as citation number 20 in the revised manuscript.

**Reviewer 2:** Eligibility Criteria - What is the rationale for exclusion of those attending hospital Emergency Departments (EDs)? Adults using these services are often considered community-dwelling, and most prescribed medicines would be filled in community pharmacies. Also are EDs not considered ambulatory care settings (see Setting)?  

**Authors: **Our rationale for excluding those attending hospital EDs is that it can be challenging to determine whether subsequent hospital admission occurs at an individual level. As we are excluding hospital based studies we feel that it is also appropriate to exclude studies based in EDs. We do accept the reviewer point that adults using these services are often considered community-dwelling and will obtain medication in community pharmacies. However, it is difficult to determine whether episodic ED visits lead to prolonged changes in the individuals prescribed medication. Furthermore, we do not consider EDs as ambulatory care settings. We view ambulatory care as clinical care provided on an outpatient basis outside the hospital setting and that does not result in a hospital admission e.g. primary care centres, outpatient clinics, day treatment clinics e.g. dialysis.

*Reference:* Medicare Payment Advisory Committee. (2021). Ambulatory Care Settings. Retrieved from http://www.medpac.gov/-research-areas-/ambulatory-care-settings.

**Reviewer 2:** Risk – I agree that it would be useful to capture data on the therapeutic indication and the side effect; however, these details are often not available in health administrative data, and so would likely only be available in case studies. While therapeutic indication will not necessarily be available, studies using health administrative data may have selected certain patient cohorts (i.e., those with hypertension, etc) to study a particular cascade – this information would be relevant to capture.  

**Authors: **We agree that for many studies it is unlikely that information will be provide on the therapeutic indication and side effect experienced. The examination of specific patient cohorts may well be explicitly stated within administrative studies and as such we have updated our data extraction proforma to include details of any *a priori *cohort selection techniques applied (see also Page 7 of manuscript).

**Reviewer 2:** Data extraction – This is a comprehensive list but suggest adding – i) time to prescribing of drug B (i.e., time from initiation of drug A to initiation of drug B) – if recorded, this information is helpful to identify a relevant time window that providers can think about/be aware of when the side effect is likely to occur, and ii) variables considered to modify the association/effect, specifically sex and gender. This information again would be helpful in considering whether there are cascades which affect women vs. men, which can help inform targeted interventions.

**Authors: **We agree that where available the timing to initiation of Drug B is valuable information and have updated the data extraction proforma to capture this information, where available. Furthermore, details of variables that modify the association, particularly sex and gender will now be extracted (Page 7). This is particularly relevant when one considers the associations between female sex, inappropriate prescribing and ADRs. Several studies have found an association between female sex and the likelihood for inappropriate prescribing (see Hill-Taylor *et al*., 2013 for a review; Ukhanova *et al*, 2021). An examination of global pharmacovigilance data found that women report more ADRs than men (Watson, Caster, Rochon, den Ruijter, 2019). Biological sex differences including differing muscle mass, body fat, volumes of distribution and metabolic enzymes have the potential to result in altered pharmacokinetic responses to medication. Social and cultural gender differences may also influence health behaviour via differences in help seeking and adherence to behavioural actions.  An international research consortium is currently embarking on a project to sex and gender differences in prescribing cascades (iKASCADE project; Sternberg *et al*., 2021) and so the extraction of data on sex and gender is highly relevant and pertinent.

*References:*

 Hill-Taylor B, Sketris I, Hayden J, Byrne S, O'Sullivan D, Christie R. Application of the STOPP/START criteria: a systematic review of the prevalence of potentially inappropriate prescribing in older adults, and evidence of clinical, humanistic and economic impact. *J Clin Pharm Ther*. 2013;38(5):360-372. doi: 10.1111/jcpt.12059

 Ukhanova M, Markwardt S, Furuno JP, Davis L, Noble BN, Quiñones AR. Are there sex differences in potentially inappropriate prescribing in adults with multimorbidity?. *J Am Geriatr Soc*. 2021;69(8):2163-2175. doi: 10.1111/jgs.17194

 Watson S, Caster O, Rochon PA, den Ruijter H. Reported adverse drug reactions in women and men: Aggregated evidence from globally collected individual case reports during half a century. *EClinicalMedicine*. 2019;17:100188. Published 2019 Oct 25. doi: 10.1016/j.eclinm.2019.10.001

 Sternberg SA, Petrovic M, Onder G, *et al.* Identifying key prescribing cascades in older people (iKASCADE): a transnational initiative on drug safety through a sex and gender lens-rationale and design. *Eur Geriatr Med*. 2021;12(3):475-483. doi: 10.1007/s41999-021-00480-w

**Reviewer 2:** I wonder about the inclusion of intentional prescribing cascades if the intent is to “support clinicians to optimize their prescribing to benefit patient care”? If cascades are started intentionally, clinicians have likely already carefully considered that benefits outweigh harms. If you wish to have an exhaustive list, I understand the desire to include both intentional and unintentional; however, I agree with reviewer 1 that stratifying results based on intent is helpful, since those that are unintentional are more amenable to deprescribing and improving patient safety.

**Authors: **We agree that intentional cascades should follow careful consideration by the prescriber on the potential harms and benefits. McCarthy, Visentin and Rochon (2019) note that appropriate prescribing cascades are always intentional. Nevertheless, even those prescribing cascades which are appropriate at the time of initiation have the potential to become problematic or inappropriate over time. The failure to reassess the cascade within an appropriate time frame may result in the continuation of a once appropriate intentional cascade which has subsequently become a problematic one. In order to expand on these nuances we feel it is important to collate all clinically relevant prescribing cascades. We agree that stratifying the results by intent will help to demarcate those cascades where deprescribing initiatives can be targeted from those intentional cascades which require a reassessment in the future so as to ensure their appropriateness.

*References:* McCarthy LM, Visentin JD, Rochon PA. Assessing the Scope and Appropriateness of Prescribing Cascades. *J Am Geriatr Soc*. 2019;67(5):1023-1026. doi: 10.1111/jgs.15800

# Peer review: Reviewer response for version 1

**DOI:** 10.21956/hrbopenres.14533.r29875

**Published:** 2021-08-09

**Authors:** Nagham J. Ailabouni

**Affiliations:** 1 Clinical and Health Sciences, Quality Use of Medicines and Pharmacy Research Centre (QUMPRC), University of South Australia, UniSA, Adelaide, SA, Australia

Thank you for the opportunity to read your work. This is interesting and worthwhile research regarding a topic that is underreported. The protocol is written well and is comprehensive. The planned systematic review addresses a pertinent gap and will hopefully give researchers and clinicians who care for people living with multimorbidity a greater appreciation for the negative health impacts of prescribing cascades. I hope you find my minor comments and suggestions useful and beneficial. 

**Method:**

- Consider changing the inclusion criteria from “prescribed medication with potential to cause a side effect” to “any medication”. This is because even medications that are taken OTC can constitute as an important contributor to prescribing cascades. If the authors believe that details of OTC medications taken will not be captured in studies that are likely to be included in the systematic review, please mention this as a rationale for only considering prescribed medication(s).

**Introduction:**

- Whilst readers in this field might be well versed with what potentially inappropriate prescribing (PIP) is, it might be useful to clarify the definition of PIP in the first paragraph. So, for example, when “Medications provide many therapeutic benefits but these must be balanced against the potential for patient harm.” is mentioned, perhaps add that potentially inappropriate medications are those with greater potential harm than possible benefit.

*Introduction, paragraph 2, line 3:*

- “multimorbidity is prevalent in those aged 65 years or older? (not younger).” The reference cited here is a cross-sectional study that found that multimorbidity in absolute terms is more prevalent in younger than 65 years. However, I still believe the first sentence should be that generally speaking multimorbidity worldwide increases with increasing age and is more prevalent as aging occurs. Then if the authors wish to include this specific point, they could specifically mention that this particular study found this finding (i.e. multimorbidity is higher in those younger than 65 years particularly if they have a lower socioeconomic status or deprivation score).

**Data extraction:**

- Another confounder to potentially look out for, is frailty. Additionally, if frailty is not reported or taken into account in included studies, this would be an interesting finding to be discussed as frailty is often a confounder that is unmeasured and unaccounted for and frailty could be associated with the occurrence of prescribing cascades. 
- Where "adjusted sequence ratio (ASR) for prescription" and "sequence symmetry analysis" are mentioned, I recommend adding some example references that used these methods.

**Strategy for data synthesis:**

- Good list of items to be extracted. Also, it might be interested the consider narratively describing the following:
  1) The period of time the prescribing cascade has likely existed. In other words, how long has the second subsequent medication been prescribed or taken by the individual?
  2) The prescriber who initiated the prescription of the second medication (if this information is available). This is because there may be differences in rates of new medications prescribed by specialists vs GPs vs other healthcare professionals.
  3) Authors might have already considered this but grouping the identified prescribing cascades as either “intentional” or “unintentional” could be interesting to readers.
  4) Details for how long the second medication is intended to be prescribed for. So, in other words, when the second medication used to treat the ADR was prescribed, did the prescriber or the individual's clinical notes mention the likely duration this medication is ought to be prescribed and if there is a plan to eventually review and consider stopping or reducing (i.e. deprescribing) this medication?

**Discussion: **

- The authors have done a good job summarising issues of prescribing in the introduction of the protocol. It may also be helpful to also briefly highlight that prescribers, specifically GPs, often face challenges prescribing for people living with multimorbidity (recommend citing this article of mine or a similar article: Ailabouni *et al*. (2016) ^1^). This will further support what the authors plan to do with the findings of this systematic review outlined in the discussion such as for example developing a decision tool to support GPs/prescribers making prescribing decisions for people with multimorbidity. 

Is the study design appropriate for the research question?

Yes

Is the rationale for, and objectives of, the study clearly described?

Yes

Are sufficient details of the methods provided to allow replication by others?

Yes

Are the datasets clearly presented in a useable and accessible format?

Not applicable

Reviewer Expertise:

Geriatric pharmacy; deprescribing; multimorbidity

I confirm that I have read this submission and believe that I have an appropriate level of expertise to confirm that it is of an acceptable scientific standard, however I have significant reservations, as outlined above.

## References

[1] : General practitioners' insight into deprescribing for the multimorbid older individual: a qualitative study. *Int J Clin Pract* .2016;70(3) : 10.1111/ijcp.12780 261-76 10.1111/ijcp.12780 26918508

# Response

**Published:** 2021-09-07

**Authors:** Ann Doherty

**Affiliations:** Royal College of Surgeons in Ireland, Ireland

Dear Dr Ailabouni,

 Thank you for reviewing our submission and for your thoughtful feedback. Below you will find our responses to the specific comments in turn. Text labelled 'Reviewer' are the comments of the reviewer. Text labelled 'Authors' are the responses of the authors.

**Reviewer:** Consider changing the inclusion criteria from “prescribed medication with potential to cause a side effect” to “any medication”. This is because even medications that are taken OTC can constitute as an important contributor to prescribing cascades. If the authors believe that details of OTC medications taken will not be captured in studies that are likely to be included in the systematic review, please mention this as a rationale for only considering prescribed medication(s).

**Authors**: Indeed, we agree that over the counter (OTC) medications constitute an important aspect of the prescribing cascade phenomenon. We opted to consider only prescribed medication(s) as we believe that a large proportion of studies likely to be included in the review will comprise of prescription sequence symmetry analysis of administrative claims data, comprised of prescription dispensing datasets. Such datasets are not likely to provide information on OTC medication use. A Dutch study comparing patient self-report medication use with data contained in the pharmacy electronic patient record found that data on non-prescription medication use was absent for 44% of study participants (Floor-Schreudering *et al.*, 2013). Similarly, pharmacist medication reconciliation and concurrent electronic medication review in a primary care clinic found a high proportion of patients (74%) had at least one discrepancy between self-reported medication use and the electronic record, with the use of OTC medication the most common type of discrepancy (Stewart & Lynch, 2012). The manuscript has been updated (Page 5- Eligibility criteria section) to explicitly mention the rationale for examining prescribed medication only.

 References: Floor-Schreudering A, Heringa M, Buurma H, Bouvy ML, De Smet PA. Missed drug therapy alerts as a consequence of incomplete electronic patient records in Dutch community pharmacies. *Ann Pharmacother*. 2013;47(10):1272-1279. doi: 10.1177/1060028013501992

 Stewart AL, Lynch KJ. Identifying discrepancies in electronic medical records through pharmacist medication reconciliation. *J Am Pharm Assoc (2003)*. 2012;52(1):59-66. doi: 10.1331/JAPhA.2012.10123

**Reviewer**: Whilst readers in this field might be well versed with what potentially inappropriate prescribing (PIP) is, it might be useful to clarify the definition of PIP in the first paragraph. So, for example, when “Medications provide many therapeutic benefits but these must be balanced against the potential for patient harm.” is mentioned, perhaps add that potentially inappropriate medications are those with greater potential harm than possible benefit.

**Authors: **We have included a sentence (Page 4, paragraph 1) as follows: “Potentially inappropriate mediations are those where the potential for harm outweighs the possible benefit for the patient”.

 Reference: Beers MH, Ouslander JG, Rollingher I, Reuben DB, Brooks J, Beck JC. Explicit criteria for determining inappropriate medication use in nursing home residents. UCLA Division of Geriatric Medicine. *Arch Intern Med*. 1991;151(9):1825-1832. PMID: 1888249

**Reviewer:** Introduction, paragraph 2, line 3:

 “multimorbidity is prevalent in those aged 65 years or older? (not younger).” The reference cited here is a cross-sectional study that found that multimorbidity in absolute terms is more prevalent in younger than 65 years. However, I still believe the first sentence should be that generally speaking multimorbidity worldwide increases with increasing age and is more prevalent as aging occurs. Then if the authors wish to include this specific point, they could specifically mention that this particular study found this finding (i.e. multimorbidity is higher in those younger than 65 years particularly if they have a lower socioeconomic status or deprivation score).

**Authors:** We agree that this section of text requires clarification. It was our intention to state that whilst the prevalence of multimorbidity increases with increasing age, it is important not to disregard the impact of multimorbidity in younger age groups (younger than 65 years) particularly for those who experience deprivation. Hence why we are examining prescribing cascades in all adults and not those aged 65 years and older. This section of text has been updated to state the predominant association between multimorbidity and increasing age (with appropriate citations). We also note that multimorbidity can also present at an earlier age for those living in socioeconomic deprivation.

 References: Salisbury C, Johnson L, Purdy S, Valderas JM, Montgomery AA. Epidemiology and impact of multimorbidity in primary care: a retrospective cohort study. *Br J Gen Pract*. 2011;61(582):e12-e21. doi: 10.3399/bjgp11X548929

 Raffaele Palladino, John Tayu Lee, Mark Ashworth, Maria Triassi, Christopher Millett, Associations between multimorbidity, healthcare utilisation and health status: evidence from 16 European countries, *Age and Ageing*, Volume 45, Issue 3, May 2016, Pages 431–435, https://doi.org/10.1093/ageing/afw044

**Reviewer:** Data extraction:

 Another confounder to potentially look out for, is frailty. Additionally, if frailty is not reported or taken into account in included studies, this would be an interesting finding to be discussed as frailty is often a confounder that is unmeasured and unaccounted for and frailty could be associated with the occurrence of prescribing cascades.

**Author response: **We agree and thank the reviewer for this suggestions. We have updated our data extraction form to consider whether frailty is reported or not within the study under the heading “Confounders accounted for in the analysis”. The “Data Extraction” section (Page 7) of the manuscript has also been updated to reflect this.

**Reviewer 1**: Where "adjusted sequence ratio (ASR) for prescription" and "sequence symmetry analysis" are mentioned, I recommend adding some example references that used these methods.

**Authors: **We agree that some example references will aid the reader to understand these methods and have thus included two examples that have used this methodology (Data extraction section, Page 7).

**Reviewer:** Strategy for data synthesis:

 Good list of items to be extracted. Also, it might be interested the consider narratively describing the following:

 1) The period of time the prescribing cascade has likely existed. In other words, how long has the second subsequent medication been prescribed or taken by the individual?

 2) The prescriber who initiated the prescription of the second medication (if this information is available). This is because there may be differences in rates of new medications prescribed by specialists vs GPs vs other healthcare professionals.

 3) Authors might have already considered this but grouping the identified prescribing cascades as either “intentional” or “unintentional” could be interesting to readers.

 4) Details for how long the second medication is intended to be prescribed for. So, in other words, when the second medication used to treat the ADR was prescribed, did the prescriber or the individual's clinical notes mention the likely duration this medication is ought to be prescribed and if there is a plan to eventually review and consider stopping or reducing (i.e. deprescribing) this medication?

**Author response: **We agree that the inclusion of these additional aspects will strengthen the data synthesis. As such as we have updated the data extraction proforma and Data Extraction section (Page 7) to include: period of time the cascade has likely existed; prescriber who initiated the second medication; duration the second medication was intended to be prescribed for; and plan to review this second medication, where available. We also agree that stratifying the identified cascades by intentionality will be helpful to readers and so we have updated the data synthesis section (Page 8) to explicitly state this.

**Reviewer:** Discussion: The authors have done a good job summarising issues of prescribing in the introduction of the protocol. It may also be helpful to also briefly highlight that prescribers, specifically GPs, often face challenges prescribing for people living with multimorbidity (recommend citing this article of mine or a similar article: Ailabouni *et al.* (2016)1). This will further support what the authors plan to do with the findings of this systematic review outlined in the discussion such as for example developing a decision tool to support GPs/prescribers making prescribing decisions for people with multimorbidity.

**Authors: **We agree that the inclusion of further detail on the challenges faced by prescribers when prescribing for those with multimorbidity and have included a brief section on this and included the suggestion citation to provide context for this (Discussion section, paragraph 2).
